# A sequence-based genetic linkage map as a reference for *Brassica rapa *pseudochromosome assembly

**DOI:** 10.1186/1471-2164-12-239

**Published:** 2011-05-14

**Authors:** Yan Wang, Silong Sun, Bo Liu, Hui Wang, Jie Deng, Yongcui Liao, Qian Wang, Feng Cheng, Xiaowu Wang, Jian Wu

**Affiliations:** 1 Institute of Vegetables and Flowers, Chinese Academy of Agricultural Sciences, Beijing, 100081, China

## Abstract

**Background:**

*Brassica rapa *is an economically important crop and a model plant for studies concerning polyploidization and the evolution of extreme morphology. The multinational *B. rapa *Genome Sequencing Project (BrGSP) was launched in 2003. In 2008, next generation sequencing technology was used to sequence the *B. rapa *genome. Several maps concerning *B. rapa *pseudochromosome assembly have been published but their coverage of the genome is incomplete, anchoring approximately 73.6% of the scaffolds on to chromosomes. Therefore, a new genetic map to aid pseudochromosome assembly is required.

**Results:**

This study concerns the construction of a reference genetic linkage map for *Brassica rapa*, forming the backbone for anchoring sequence scaffolds of the *B. rapa *genome resulting from recent sequencing efforts. One hundred and nineteen doubled haploid (DH) lines derived from microspore cultures of an F1 cross between a Chinese cabbage (*B. rapa *ssp. *pekinensis*) DH line (Z16) and a rapid cycling inbred line (L144) were used to construct the linkage map. PCR-based insertion/deletion (InDel) markers were developed by re-sequencing the two parental lines. The map comprises a total of 507 markers including 415 InDels and 92 SSRs. Alignment and orientation using SSR markers in common with existing *B. rapa *linkage maps allowed ten linkage groups to be identified, designated A01-A10. The total length of the linkage map was 1234.2 cM, with an average distance of 2.43 cM between adjacent marker loci. The lengths of linkage groups ranged from 71.5 cM to 188.5 cM for A08 and A09, respectively. Using the developed linkage map, 152 scaffolds were anchored on to the chromosomes, encompassing more than 82.9% of the *B. rapa *genome. Taken together with the previously available linkage maps, 183 scaffolds were anchored on to the chromosomes and the total coverage of the genome was 88.9%.

**Conclusions:**

The development of this linkage map is vital for the integration of genome sequences and genetic information, and provides a useful resource for the international *Brassica *research community.

## Background

The genus *Brassica *is one of the core genera within the tribe *Brassicea*. It comprises a large number of crops with a wide spectrum of morphological variation that can be cultivated under a variety of agro-climatic conditions. *Brassica*s provide vegetable oil, fresh and preserved vegetables, fodder and condiments, as well as being important sources of dietary fibre, vitamin C and nutritionally beneficial factors including anti-cancer compounds [1]. There are six representative species in the *Brassica *genus including three diploid species *B. rapa *(AA, 2n = 20), *B. nigra *(BB, 2n = 16) and *B. oleracea *(CC, 2n = 18), and three amphidiploids *B. juncea *(AABB, 2n = 36), *B. napus *(AACC, 2n = 38) and *B. carinata *(BBCC, 2n = 34). The genetic relationships among these *Brassica *species are well defined in U's triangle [2]. One of the diploid species, *B. rapa*, comprises a variety of morphologically diverse cultivated types including Chinese cabbage, misuna, aburana, flowering cabbage, turnip, turnip rape, yellow sarson, tatsoi and komatsuna, and these provide leaf heads, leaves, flowering stems, turnips and seeds, the productive organs for economical consumption [3,4]. Furthermore, *B. rapa *is an excellent model for studying polyploidy genome evolution owing to its paleohexaploid ancestry and its close evolutionary relationships with *Arabidopsis thaliana *[5].

The multi-national *B. rapa *Genome Sequencing Project (BrGSP) was launched in 2003 owing to the economical and biological importance of *B. rapa*, and the A3 chromosome was sequenced using traditional Sanger technology [5]. In 2008, rapid next generation sequencing technology was employed for *B. rapa *genome sequencing and a high density genetic map based on sequence-tagged markers is necessary to anchor the assembled scaffolds to chromosomes. Several maps concerning *B. rapa *have been published to be used as reference genetic maps for pseudochromosome assembly (http://www.brassica-rapa.org) [6-8] despite their coverage only allowing in total 73.6% of the scaffolds being anchored on to chromosomes [The *Brassica rapa *Genome Sequencing Project Consortium: The genome of the mesohexaploid crop species *Brassica rapa*, submitted].

Genetic mapping is important for understanding the origin of and relationships among the genomes of *Brassica *species. Genetic linkage maps can also provide improved insight into genome organization and evolution through comparative mapping, and serve as the basis for genetic studies concerning various agronomic traits through the localization of major genes and quantitative trait loci (QTLs). Furthermore, they can aid breeding programs with the development of marker assisted selection (MAS) [9]. More than 20 genetic linkage maps have been constructed for *B. rapa *using a range of marker types including Restriction Fragment Length Polymorphisms (RFLPs), random amplified polymorphic DNA (RAPD), amplified fragment length polymorphisms (AFLPs), sequence-related amplified polymorphisms (SRAPs) and simple sequence repeats (SSRs) [6-8,10-20]. However, there are limited published data concerning sequence-tagged PCR-markers, predominantly SSRs, mapped in *B. rapa *[6-8,15,19], particularly markers that could provide anchors for the *B. rapa *genome that are transferable to other mapping populations.

Recent developments in sequencing technology have simplified and accelerated the discovery of sequence variants, enabling development of sequence-based markers including single nucleotide polymorphisms (SNPs) and insertion/deletion polymorphisms (InDels) [21]. InDels and SNPs are the markers of choice for high-resolution genetic mapping and association studies owing to their abundance and distribution throughout the genome [22,23]. For example, a study investigating genetic variation on human chromosome 22 suggested that InDels represent 18% of the polymorphisms on this chromosome [24]. Studies concerning genetic variation in *A. thaliana *have demonstrated that InDels represent 34% of all genetic polymorphisms [25]. Furthermore, InDels can contribute directly to a phenotype [26], or can associate with a phenotype as a result of linkage disequilibrium [27]. By re-sequencing 1,398 sequence-tagged sites (STSs) in eight *B. rapa *genotypes, Park et al. identified and characterized 6,753 InDels in the gene space of the *B. rapa *genome [28]. InDel polymorphisms are the second most frequent type of polymorphism in the genome, and can be genotyped using simple procedures including the analysis of size polymorphisms of polymerase chain reaction (PCR) products on agarose gels [29,30]. Another advantage of InDel markers is the improbability of two InDel mutations being exactly the same length and at the same genomic position. Therefore, shared InDels represent identity-by-descent [31]. Very few InDel markers have been used to construct genetic linkage maps of *B. rapa*. With the recent completion of the sequencing of the *B. rapa *genome [The *Brassica rapa *Genome Sequencing Project Consortium: The genome of the mesohexaploid crop species *Brassica rapa*, submitted], the development of whole genome-wide InDel markers based on re-sequencing has become feasible, and this will be a useful resource for the international research community.

In this study, InDel and SSR markers, both of which are sequence-tagged PCR markers, were used to construct a high resolution genetic map of *B. rapa*. The map was used as a reference linkage map to anchor and orient sequence scaffolds for *B. rapa *genome assembly.

## Results

### Generation of markers and polymorphism survey

The process of genotyping InDel polymorphisms was optimised. A range of annealing temperatures from 55°C to 63°C for 16 primer pairs were tested, and the results demonstrated that annealing at 57°C produced favourable results for all primer pairs. Therefore, amplification of InDels was accomplished using a single, uniform set of conditions with a denaturing temperature of 57°C. The size of amplified DNA fragments was within the range of 80-200 bp and 4-10 bp insertion/deletions were used as markers. Therefore, the PCR products could be separated using PAGE (polyacrylamide gel electrophoresis).

A population named RCZ16_DH with 119 doubled haploid (DH) lines derived from an F1 cross between a Chinese cabbage DH line (Z16) and a rapid cycling inbred line (L144) was used for genetic map construction. To construct the RCZ16_DH map, 520 unique PCR-based InDel markers for 'Z16' and 'L144' were designed. Of these, 427 (82.1%) yielded single PCR fragments and demonstrated polymorphism, eight (1.6%) did not amplify any products and 85 (16.3%) had no polymorphism. Among the 427 polymorphic InDels, 411 yielded single PCR products with different lengths for the two parental lines, while 16 had amplicons in one of the parents only. These 16 primer pairs were discarded to prevent false negatives when carrying out PCR. An additional 333 InDel markers were screened, and 163 were designed on the basis of the InDel variations between 'Chiifu-401-42' and 'Kenshin'; 170 pairs were designed on the basis of variations between 'Chiifu-401-42' and 'L144'. In total, 415 polymorphic InDel primer pairs including 323 from 'Z16' and 'L144', 40 from 'Chiifu-401-42' and 'Kenshin', and 52 from 'Chiifu-401-42' and 'L144', were scored and used to generate the RCZ16_DH genetic linkage map using 119 DH lines.

For assessment of SSRs, 1309 SSRs from a range of sources were tested [6,7,16,32-37]; 130 presented with polymorphic banding patterns between the parental lines and 92 easily scored SSRs were screened for the 119 DH lines. Of these, three SSR marker assays (BoE347, BoE974 and KBRH139B23) detected more than one segregating locus. The information concerning all mapped InDel and SSR loci is presented in Additional File 1.

### Construction of the RCZ16_DH linkage map

**Figure 1:**
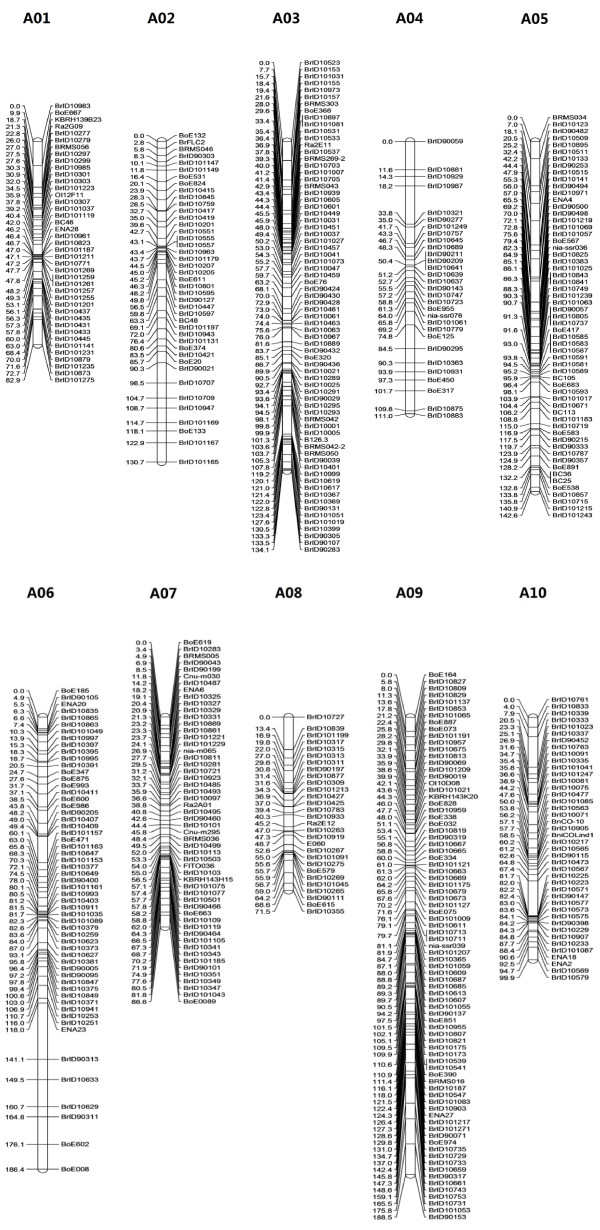
**Linkage map of *B. rapa *L. ssp. *pekinensis***. Recombination distances are indicated on the left hand side of each linkage group in centimorgans (cM), and the locus names are depicted on the right side of each linkage group. The map demonstrates the distribution of 507 loci along ten linkage groups (A01-A10).

A total of 507 markers including 415 InDel markers and 92 SSR markers were assigned to 10 linkage groups (Figure 1) and designated as A01-A10, corresponding to the previously published linkage maps [6,8,15,19,20,38]. Each of the ten linkage groups contained at least two previously published SSR markers that provided anchors to previously published maps, with the exception of chromosome A04 (Additional File 1). Anchoring A04 by the two InDels (BrID90277 and BrID10363) located at the scaffolds on which the two BACs (KBrB068A13 and KBrB033O04) were positioned was confirmed using three SSR markers, locating at A04 in the maps of VCS_DH, JWF3P and CKDH (Additional File 1). The linkage map covered a genetic distance of 1234.2 cM, with an average distance of 2.43 cM between ordered adjacent markers (Table 1). The largest linkage group contained the maximum number of markers (81) for A09 and spanned 188.5 cM, while the smallest contained the minimum number of markers (29) for A08, with a length of 71.5 cM. The distribution of InDels along the linkage groups ranged from 25 on A04 and A08 to 65 on A09, and the distribution of SSRs ranged from 3 on A10 to 16 on A09. The physical length of the *B. rapa *genome is approximately 283.8 Mbp [The *Brassica rapa *Genome Sequencing Project Consortium: The genome of the mesohexaploid crop species *Brassica rapa*, submitted]. The map defined herein represents average genetic and physical intervals of 2.43 cM and 559.8 Kbp per marker, respectively. Therefore, it is currently the most saturated linkage map for *B. rapa*. In the final linkage map, 90% of the covered genome had a marker within 5 cM. However, there were still two gaps (>15 cM) [7] of 23 cM and 16 cM on A06 and A04, respectively.

**Table 1:** Summary of the genetic linkage map of *B. rapa *constructed using sequence-based markers

Linkage group	Length (cM)	Markers (no.)	Density (cM/marker)	**Intervals**^**a **^**(no.)**	**Gaps**^**b **^**(no.)**	Ave. interval (cM)
A01	82.9	41	2.02	28	0	2.96
A02	130.7	42	3.11	34	0	3.84
A03	134.1	72	1.86	41	0	3.27
A04	111.0	30	3.70	27	1	4.11
A05	142.6	59	2.42	35	0	4.07
A06	186.4	57	3.27	39	1	4.78
A07	86.6	54	1.60	36	0	2.41
A08	71.5	29	2.46	20	0	3.57
A09	188.5	81	2.33	59	0	3.20
A10	99.9	42	2.38	26	0	3.84
Total	1234.2	507	2.43	345	2	3.61

^a ^Adjacent markers > 1 cM; ^b ^Interval distance between adjacent markers ≥15 cM [7].

### Alignment of the linkage groups to *B. rapa *pseudochromosomes

The high-resolution RCZ16_DH genetic linkage map with 507 sequence-based markers was successfully used to anchor and orientate scaffolds for the genome assembly of *B. rapa *together with the three publicly available *B. rapa *genetic maps, VCS_DH, JWF3P (http://www.brassica-rapa.org) and CKDH [6-8]. The markers of the RCZ16_DH genetic linkage map were aligned to the *B. rapa *genome sequence using their primer sequences; 66 SSR markers and the 415 InDel markers were mapped to a total of 152 scaffolds. Among these 481 positioned markers, three SSR markers (BoE347, BoE974 and KBRH139B23) were detected on more than one segregating locus. Therefore, only 478 unique markers were used for anchoring 152 scaffolds of *B. rapa*, covering 82.9% of the assembled genome. The uniquely aligned markers used to anchor scaffolds ranged from 76 for A09 to 27 for A04. The number of scaffolds anchored on to the chromosomes by these markers ranged from 6 for A10 to 32 for A09. In 417 cases, more than one marker was located on a single scaffold, allowing 91 scaffolds to be oriented throughout the 10 chromosomes. The details of the markers used to anchor the scaffolds are presented in Table 2.

**Table 2:** Summary of sequence-based markers used to anchor and orient the scaffolds to linkage groups of *B. rapa*

Linkage group	Markers (no.)	InDels (no.)	SSRs (no.)	Anchored InDels (no.)	Anchored SSRs (no.)	Anchored Scaff. no.	Anchored **Scaff.lg (bp)**^**a**^	**Anchored Scaff.cv (%)**^**b**^
A01	41	34	7	34	5	21	20867750	7.35
A02	42	32	10	32	8	18	27594208	9.72
A03	72	61	11	61	9	9	31313061	11.03
A04	30	25	5	25	2	14	16383005	5.77
A05	59	46	13	46	8	17	21627037	7.62
A06	57	46	11	46	8	14	25300054	8.91
A07	54	42	12	42	9	10	21622174	7.62
A08	29	25	4	25	3	11	16765946	5.91
A09	81	65	16	65	11	32	36142156	12.73
A10	42	39	3	39	3	6	17594535	6.20
Total	507	415	92	415	66	152	235209926	82.86

^a ^anchored scaffold length; ^b ^anchored scaffold coverage.

To compare the RCZ16_DH map to the three publicly available genetic linkage maps, VCS_DH (354 markers), JWF3P (498 markers) and CK_DH (719 markers), the BACs where the markers were located were used as the genetic loci. These BACs were aligned to scaffold sequences and regarded as common genetic loci if they were within a range of 100 Kbp in distance to the position of InDels or SSRs on the RCZ16_DH map. Two hundred (39.4%) of the 507 markers on the RCZ16_DH map located common loci on at least one of the three previously published maps. Using VCS_DH, JWF3P and CK_DH maps, there were 84 (171 Mb), 97 (185 Mb) and 91 (175 Mb) scaffolds anchored to the corresponding chromosomes, respectively. Combining these three maps, the total coverage was 73.6% of the *B. rapa *genome. However, when taken together with the RCZ16_DH map, the anchored scaffold number and the total coverage of the *B. rapa *genome increased to 183 and 88.9%, respectively (Table 3).

**Table 3:** Information relating to scaffold anchoring using four linkage maps of *B. rapa*

	RCZ16_DH	VCS_DH	JWF3P	CK_DH	Total
Marker No.	507	354	498	719	2078
Sequenced-based marker No.	507	354	498	428 (191)^a^	1787(1550)^a^
Anchored scaffold No.	152	84	97	91	183
Oriented scaffold No.	91	55	62	54	114
Physical distance (Mb)	235	171	185	175	252

^a ^The number of markers with genetic distance publicly available.

**Figure 2:**
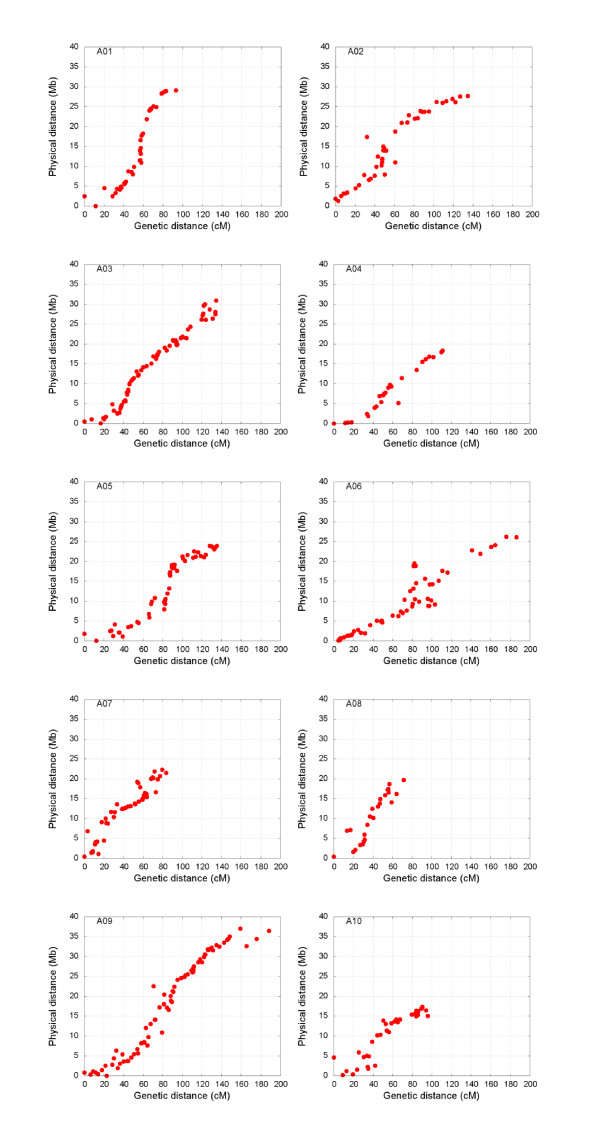
**RCZ16_DH genetic map versus physical distance map of the ten *B. rapa *pseudochromosomes**. The pseudochromosomes were constructed using markers from four genetic maps namely RCZ16_DH, VCS_DH, JWF3P (http://www.brassica-rapa.org) [6] and CK_DH [7,8].

Alignment of the RCZ16_DH linkage map with the constructed pseudochromosomes verified the accuracy of the scaffold order and orientation (Figure 2). The InDel markers were developed from re-sequencing data, and were selected to develop InDel markers from scaffold regions where there had been no marker in previous linkage maps. As a result, the map enhanced the evaluation of the quality of sequence assembly. The majority of the markers (93%) were collinear with the sequence assembly. In several assembly iterations, scaffold misplacement was visible as discontinuity or negative slopes.

## Discussion

This study concerns the comprehensive linkage analysis of *B. rapa*. The map spans 1234.2 cM and is divided into ten linkage groups corresponding to the number of *B. rapa *chromosomes, with an average distance of 2.43 cM between adjacent markers. A conspicuous characteristic of the present map is that 415 markers, accounting for 81.7% of the total mapped markers, are novel InDel markers, which increases the number of sequence-based markers for *B. rapa*.

One of the main purposes of this linkage map is to support the *B. rapa *genome sequencing project to anchor and orient scaffolds onto the chromosomes. Using publically available genetic linkage maps for *B. rapa *including VCS_DH (354 markers), JWF3P (498 markers) (http://www.brassica-rapa.org) [6] and CK_DH (313 markers and 719 markers) [7,8], 84 (171 Mb), 97 (185 Mb) and 91 (175 Mb) scaffolds were anchored to chromosomes, respectively. However, selecting markers that were evenly distributed along the genome and complementary to the previously reported maps, using the RCZ16_DH map, 152 (235 Mb) scaffolds were anchored on to the chromosomes. The RCZ16_DH map alone covers more than 82.9% of the *B. rapa *genome, indicating the potential of this sequence-based linkage map for linkage and QTL analysis.

In *B. rapa*, polymorphic DNA loci are relatively frequent. Park et al. demonstrated that the frequency of SNPs and InDels were 15.3 SNPs/kb and 4.83 InDels/kb by resequencing 1,398 STSs based on the 557 BAC sequences of *B. rapa *ssp. *pekinensis *cv Chiifu-401-42 [28]. Among the 28,222 sequence variants of *B. rapa*, approximately 24% were InDels. This high frequency of InDels has also been reported for other species including maize [39,40], sugar beet [41], barley [42] and Arabidopsis [25]. In the present study, PCR-based InDel markers were developed using 80 to 200 bp PCR products, and the insertion/deletion size varied between 4 to 10 bp. The InDel polymorphisms were genotyped using a simple procedure that analyzed size polymorphisms of PCR products.

The diploid *Brassica *genomes contain large replicated blocks of collinear segments within and between linkage groups. These are thought to have derived from a polyploid ancestor, although the exact mechanism by which this occurred is debatable [43]. In the linkage map generated herein, only three SSR markers detected multiple loci and no evidence of conserved blocks of synteny can be deduced from this. This low level of detection of the replication within the genome could be due to the marker types used to construct the map. The InDel markers were developed directly from scaffold sequences by selecting unique InDels, and ambiguous markers were excluded from the marker data set. Furthermore, SSR markers are usually located in non-coding sequences, which are less well conserved between replicated blocks than coding regions.

A total of 92 SSR markers are present in the map developed in this study, and 66 of these could be aligned to the scaffold sequences using stringent criterion of 100% match of the primer sequences. Among the 26 unmapped markers, 20 were designed on the basis of EST sequences of *B. oleracea *(BoE set and Ol set). For these markers, mismatches could exist within the primer sequences preventing them from being mapped to the scaffold sequence of *B. rapa*. Five of the six unmapped SSRs derived from *B. rapa *sequences were mapped to scaffolds with only one side primer, and this could be due to the gaps in the sequence assembly.

Alignment of the RCZ16_DH map to pseudochromosomes indicated high collinearity between the genetic and physical distance (Figure 2). However, the alignment result demonstrated that there were outliers distributed on the linkage groups A02, A04, A06, A08 and A10. This could be due to scaffolds being too small or the genomic region having too little recombination to allow precise placements or orientations, as a relatively small mapping population was used in the present study. The small population size could also lead to the relatively high statistical errors when there were missing marker data for some DH lines. The other possible reason for the inconsistency of genetic and physical distances could be assembling errors as the InDels were designed on the basis of the assembled scaffold sequences.

## Conclusions

This study describes the use of sequence-based and highly polymorphic InDel markers to construct a highly resolute reference genetic map of *B. rapa*. The result is an improved resource for fine mapping of quantitative trait loci, identifying candidate genes and map-based gene isolation.

## Methods

### RCZ16_DH mapping population

The RCZ16_DH mapping population of 119 doubled haploid (DH) lines was derived from a cross between a DH Chinese cabbage (*Brassica rapa *ssp. *pekinensis*) line (Z16) and an inbred rapid cycling line (L144). A wide range of variation exits in terms of morphological traits among the individual lines. DNA from the parents and the DH plants was isolated from mature leaves as described by Wang et al. [44].

### Molecular marker analysis

#### InDel markers

The L144 and Z16 lines were re-sequenced using Illumina GAII with depths of 40 X and 2.5 X genome coverage, respectively. In addition, a Chinese cabbage line, Kenshin, was re-sequenced at 0.1 X using 454 sequencing technology (provided by Dr. David Edwards). InDels were detected by the alignment of reads to reference sequences [31]. The 4-10 bp insertion/deletions were used to develop markers. Primer 3 online software (Whitehead Institute, Cambridge, MA) was used to design primers for amplification of InDels. The criteria used for designing the primers included the following: (1) the amplified DNA fragments were within the range 80-200 bp; (2) Tm ranged from 55 to 63°C, and the difference in Tms within a primer pair was less than 3°C; (3) the GC content was greater than 35%. Each PCR was performed in a 15 μl reaction volume containing 0.4 units of *Taq *DNA polymerase with 1 × PCR buffer (Tiangen, Beijing, China), 0.5 μM of each primer, 300 μM of each dNTP, 1.5-2.0 mM MgCl_2 _and approximately 30 ng genome DNA as templates. Thermocycling began at 94°C for 7 min, followed by 35 cycles of 94°C for 40 s, 57°C for 40 s and 72°C for 1 min, and a final extension at 72°C for 10 min. PCR products were separated on 8% polyacrylamide gels and visualized using silver staining.

#### SSR markers

A total of 1309 SSR markers [6,7,15,32-37] were used to screen for polymorphisms between the two parental lines. The PCR reaction was same as that used for InDels. Thermocycling began at 95°C for 10 min, followed by 35 cycles of 94°C for 1 min, 55°C for 1 min, 72°C for 1.5 min and a final extension at 72°C for 10 min before holding at 12°C. PCR products were separated on 8% polyacrylamide gels and visualized using silver staining.

### Linkage map construction

Linkage analysis and genetic map construction were performed using JoinMap 4.0 software (http://www.kyazma.nl) [45]. Initial linkage groups were established on the basis of a LOD value ≥ 7 and the Haldane's [46,47] mapping function was used to convert recombination data into map distances.

### Alignment of linkage groups to the physical map

To reconile the linkage groups with the ten *B. rapa *chromosomes, the genetic map was aligned to the pseudochromosomes [The *Brassica rapa *Genome Sequencing Project Consortium: The genome of the mesohexaploid crop species *Brassica rapa*, submitted] on the basis of the primer sequence of the markers. The InDel markers were developed directly from scaffold sequences, and the SSRs were considered anchored if the sequence of both primers matched the scaffold sequence perfectly.

## Authors' contributions

YW generated the InDel markers, analyzed marker data and drafted the manuscript. SS carried out linkage analysis and anchoring of scaffolds to the linkage map and compared the genetic map with a physical map. BL analyzed the re-sequencing data and extracted all InDel positions and designed the InDel primers. HW, JD and YL participated in the InDel marker survey. WQ generated all SSR markers. FC participated in comparative analysis of the genetic map with the physical map. WX designed the study and participated in coordination of the study. WJ participated in linkage analysis and coordinated the study. All authors read and approved the final manuscript.

## Supplementary Material

Additional file 1 **Details of the 507 sequence-based markers on the RCZ16_DH map, and information relating to anchored scaffolds using the RCZ16_DH map and the other three publicly available genetic linkage maps**. Click here for file
